# Performance of *Trichogramma evanescens* Westwood (Hymenoptera: Trichogrammatidae) on *Spodoptera frugiperda* (J.E. Smith) (Lepidoptera: Noctuidae) eggs at laboratory and field conditions

**DOI:** 10.1038/s41598-024-77347-0

**Published:** 2024-11-05

**Authors:** Hosam M. K. H. El-Gepaly, Khaled M. A. Abdelhameed, Shimaa Y. E. Shakl, Ahmed A. Saleh, Elsayed E. Hafez

**Affiliations:** 1 Biological Pest Control Research Department, Plant Protection Research Institute, Agricultural Research Center, Giza, Egypt; 2 Apicalture Department, Plant Protection Research Institute, Agricultural Research Center, Giza, Egypt; 3 Department of Plant Protection, Faculty of Agricultural and Natural Resources, Aswan University, Tingar, Aswan, Egypt; 4 City of Scientific Research and Technology Applications, Arid Lands Cultivations Research Institute, New Borg El-Arab, 21934 Alexandria Egypt; 5 Animal and Fish Production Department, Faculty of Agriculture (Al-Shatby), Alexandria University, Alexandria City, 11865 Egypt

**Keywords:** *Trichogramma evanescens*, *Spodoptera frugiperda*, Fall armyworm, FAW, Biological aspects, Biological techniques, Developmental biology, Ecology, Plant sciences, Environmental sciences

## Abstract

The fall armyworm (FAW), *Spodoptera frugiperda* (Smith), is a significant pest threatening crops like maize across Africa, necessitating sustainable pest management alternatives. This study evaluates the efficacy of *Trichogramma evanescens* as a biological control agent against FAW egg masses in Egypt under laboratory and semi-field conditions. FAW larvae were initially collected from infested maize fields and reared on castor-oil plant leaves. Meanwhile, *T. evanescens* was propagated using *Sitotroga cerealella* eggs as hosts. The host eggs, aged 18 to 24 h, were sterilized with UV light to prevent host development while maintaining suitability for parasitism. Using custom-designed parasitoid incubators and hemisphere clip-cages, experiments focused on various egg mass configurations, assessing the effects of scales and layering. Laboratory conditions were controlled at 25 ± 2ºC and 55 ± 5% relative humidity, while semi-field trials used large cages in maize fields to approximate natural conditions. The results showed that in laboratory settings, parasitism rates averaged 5.96%, 2.00%, and 1.56% for non-, average-, and dense-scale egg masses, respectively. For egg masses with varying layers, parasitism rates were 5.24% for single-layer, 3.09% for double-layer, and 1.18% for ple-layer, regardless of scale presence. In semi-field conditions, parasitism rates were 1.01% for single-layer, 1.13% for double-layer, and 0.59% for triple-layer egg masses. Correspondingly, parasitism rates for non-, average-, and dense-scale eggs were 1.85%, 0.60% and 0.27%. The study concludes that *T. evanescens* shows promise for integrated pest management programs; however, its effectiveness is constrained by physical and environmental variables. Optimizing the timing of parasitoid releases and selecting robust strains could enhance the effectiveness of biological control, reducing reliance on chemical pesticides in Egypt.

## Introduction

The fall armyworm (*Spodoptera frugiperda* J.E. Smith) (Lepidoptera: Noctuidae) has gained notoriety as a formidable invasive pest, first recorded in West and Central Africa in early 2016. The pest’s rapid expansion throughout the continent, reaching Upper Egypt’s Aswan Governorate by May 2019, poses significant challenges to agriculture due to its voracious appetite for crucial crops, particularly maize^1–6^. This expansion is facilitated by the insect’s remarkable adaptability to diverse climatic conditions and its ability to migrate over long distances, making containment efforts complex.

Initial studies conducted by Gamil^7^, Dahi et al.^8^. , and Abd Elmageed et al.^9^. in Egypt have focused on the insect’s biological and thermal characteristics under regulated conditions. These pioneering studies laid the groundwork for developing effective management strategies tailored to Egypt’s unique agricultural ecosystem. The findings underscored the urgent need for integrated management approaches^10–13^, given the pest’s propensity to cause substantial economic losses by directly reducing maize yield and quality^14^.

Globally, measures to combat *S. frugiperda* have predominantly relied on chemical interventions, yet the effectiveness of such methods is waning due to the development of pesticide resistance. Countries such as Ethiopia and Kenya have experienced the limited success of pesticides, compounded by environmental concerns regarding toxic residues and impacts on non-target species^15,16^. This scenario has driven the search for sustainable alternatives, particularly biological control agents.

In the realm of biological control, the exploration of natural enemies of FAW has identified several promising candidates, particularly egg and larval parasitoids^9,17–22^. The parasitoids *Telenomus* sp. and *Trichogrammatoidea* sp. have been the focus of extensive studies aimed at assessing their efficacy in targeting FAW eggs^23^. The genus *Trichogramma* has gained widespread recognition globally for its application in managing Lepidopteran pests across various crops. Despite its broad use, *Trichogramma evanescens* (Westwood), extensively employed in Egypt for controlling various lepidopteran pests, has not been rigorously tested against FAW, highlighting a significant gap in current research^24,25^.

Integrating *T. evanescens* into the Integrated Pest Management (IPM) framework offers a promising, eco-friendly approach for controlling *S. frugiperda*. The use of biological control aligns with global sustainability goals and offers a dual benefit of reducing chemical pesticide reliance while potentially enhancing crop resilience. However, to optimize the use of *T. evanescens*, it is crucial to conduct detailed investigations into its parasitism rates and behavioral patterns when exposed to FAW eggs. Such studies are vital for understanding its interactions within the complex agro-ecosystem and for tailoring IPM strategies that address specific pest dynamics^26,27^.

This research aims to evaluate the efficacy of *T. evanescens* against FAW eggs through comprehensive laboratory and field trials. By investigating parasitism rates across different egg mass configurations, this study intends to contribute significantly to refining pest control strategies that mitigate the economic impact of *S. frugiperda* on Egypt’s agricultural productivity, supporting sustainable food security initiatives. Moreover, these insights hold potential applicability to broader African contexts facing similar pest challenges.

## Materials and methods

### Mass production of the fall armyworm

FAW larvae were collected from infested maize fields in early October 2022 at the Kom-Ombo Research Station, Aswan, Egypt. The larvae were adapted to rearing on the leaves of castor-oil plants (*Ricinus communis* L.) in the laboratory at the Plant Protection Research Institute, Kom-Ombo Research Station (GPS: Latitude 24° 28’ 9” N, Longitude 32° 55’ 10” E), under controlled room conditions (25 ± 2ºC and 55 ± 5% relative humidity). Newly hatched larvae were transferred immediately upon hatching to avoid predation on the remaining eggs. Early instars were supplied with small pieces of castor-oil leaves, while later instars were provided with whole leaves. Groups of 10 larvae at the same instar were reared together in glass jars (2-liter capacity) covered with fine mesh fabric. The castor-oil leaves were replaced daily, and the jars were cleaned until pupation. Near pupation, a thin layer of fine sand was spread on the bottom of each glass jar to facilitate successful pupation. FAW larvae are reared in transparent cages under controlled conditions, maintaining a stable temperature of 25 ± 2ºC and humidity of 55 ± 5%. Fresh castor-oil leaves are provided daily, with meticulous cleaning to ensure hygiene and prevent disease. As larvae transition to pupation, a sand substrate is introduced to facilitate successful pupation. Subsequently, pupae are collected, aged, and placed in wooden frames with wire mesh side cages measuring 50 cm³. After emerging, adults are housed in larger mesh cages, where a soaked cotton ball in a 10% sugar solution is hung to nourish them. Additionally, oleander branches are provided for egg-laying. Five terminal branches of *Nerium oleander* were inserted into glass vials filled with water to maintain the vitality and freshness of the branches for oviposition. The branches were replaced daily with fresh ones, and the leaves with attached eggs were cut and used either for rearing the colony (only on castor-oil leaves) or for conducting parasitism experiments.

### Rearing *Trichogramma evanescens*

The rearing process for *Trichogramma evanescens* involves several meticulous steps, beginning with the collection and initial rearing of fall armyworm (FAW) larvae from maize fields; (a) Collection and Initial Rearing: FAW larvae were collected from maize fields in Aswan, Egypt, and adapted to laboratory conditions using castor-oil plant leaves at the Kom-Ombo Research Station. Larvae were maintained under controlled conditions of 25 ± 2ºC and 55 ± 5% relative humidity. (b) Larval Care: Newly hatched larvae were immediately transferred to prevent predation. Early instars received small leaf pieces, while later instars were offered whole leaves. Ten larvae were housed in 2-liter glass jars with daily leaf replacement and cleaning. (c) Pupation Process: For pupation, a thin sand layer was added to the jars to support successful pupation. Once pupated, larvae were moved to transparent cages with consistent temperature and humidity control. (d) Adult Maintenance: Emerged adults were housed in mesh cages with a cotton ball soaked in a 10% sugar solution for nutrition. Oleander branches were provided for egg laying, replaced daily to maintain freshness.

### Mass production of *Trichogramma evanescens*

A colony of *Sitotroga cerealella* (factitious host) was routinely reared in the Bio-Lab at Shandaweel, following the protocols outlined by Grimm and Lawrence^28^ and Abdel-Hameid^29^. The insects were reared on wheat grain. Host eggs, aged 18 to 24 h, were then sterilized using ultraviolet (UV) light (TUV T8 UV-C Lamp, Philips Lighting, Netherlands) to prevent their further development while maintaining their condition for optimal suitability in parasitism. These sterilized eggs were collected, glued onto Tricho-cards, and placed into plastic jars, where they were exposed to parasitoids and maintained in the laboratory at 25–28 °C for 24 h. They were then either released into the fields or stored at 10 °C before field releases. In this experiment, non-glued host eggs were used in the parasitism process using handmade small parasitoid incubators (PI). These incubators were developed to maintain multiple ages of parasitized host eggs, enabling a sustained colony of multi-age adult parasitoids to assess the efficiency of parasitism by *T. evanescens* on newly laid FAW eggs.

### Parasitoid incubator (PI)

**Fig. 1:**
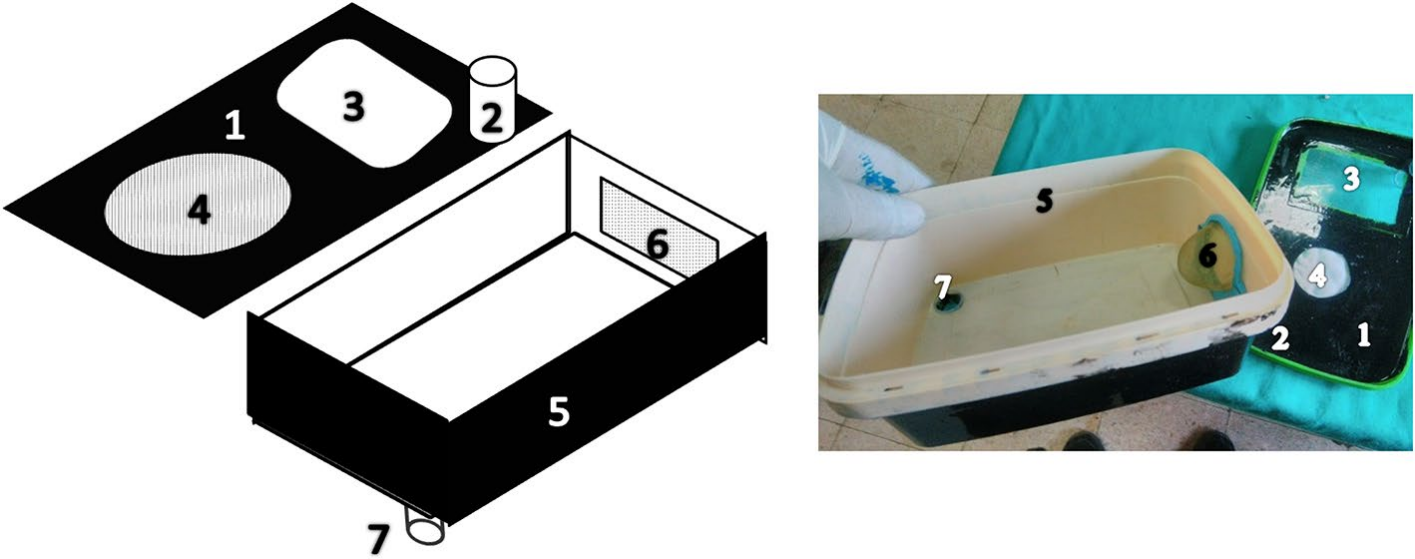
The parasitoid incubator (PI) components are 1- Top cover, 2- Entrance hatch, 3- Transparent observation pane, 4- Fabric-covered ventilation hole, 5- Main tray, 6- Chiffon hemisphere for parasitoid performance, and 7- Exit hatch.

The main objective is to consistently provide adult parasitoids of multiple ages to ensure they encounter FAW eggs immediately after they are laid. To achieve this, a handmade device, referred to as a parasitoid incubator (PI) (Fig. 1), was designed to house parasitoids of various ages along with freshly laid FAW eggs. Grain moth eggs were used as hosts during the rearing of *Trichogramma* for the experiment. In this setup, the host eggs are not attached to a paper sheet as usual; instead, fresh host eggs are placed daily into an upper slot, while parasitized eggs are harvested from a designated lower slot. A drop of honey is placed on the wall of the device to feed the adult parasitoids (Fig. 1). This method allows for the daily collection of parasitized eggs and enables the mixing of different ages of parasitoids to produce continuous waves of adult parasitoids.

### Laboratory parasitism test

To conduct the parasitism experiment, egg masses were obtained from the FAW colony twice daily. Each egg mass, including a part of the oleander leaf, was segmented and tied with a sewing thread. Each egg mass was labelled with the date, scale condition after stereomicroscope examination (classified as without, average, or dense scales), and an estimated number of eggs. The number of FAW eggs in multilayer egg masses was calculated by multiplying the number of eggs in the upper layer by the number of layers, then adding the marginal eggs^30^. Based on these calculations, 1.3 to 3.6 adult parasitoid individuals were used per FAW egg. The egg masses were categorized into three groups: small (fewer than 100 eggs), medium (101–200 eggs), and large (more than 200 eggs). Each FAW egg mass tied to the thread was inserted through the entrance hatch of the PI (Fig. 1). The PIs had been pre-supplied with 50, 100, and 150 parasitized *S. cerealella* eggs per day, corresponding to the estimated FAW egg masses. The PIs were prepared immediately upon the emergence of FAW adults. Each FAW egg mass remained in the PI for one day, after which the egg masses were transferred to plastic vials (2* × *7 cm) maintained at the required temperature (33.4 ± 1.12ºC) and relative humidity (66.5 ± 3.2%). Daily observations were conducted to record parasitism and remove hatched larvae.

### Semi-field experiment

**Fig. 2:**
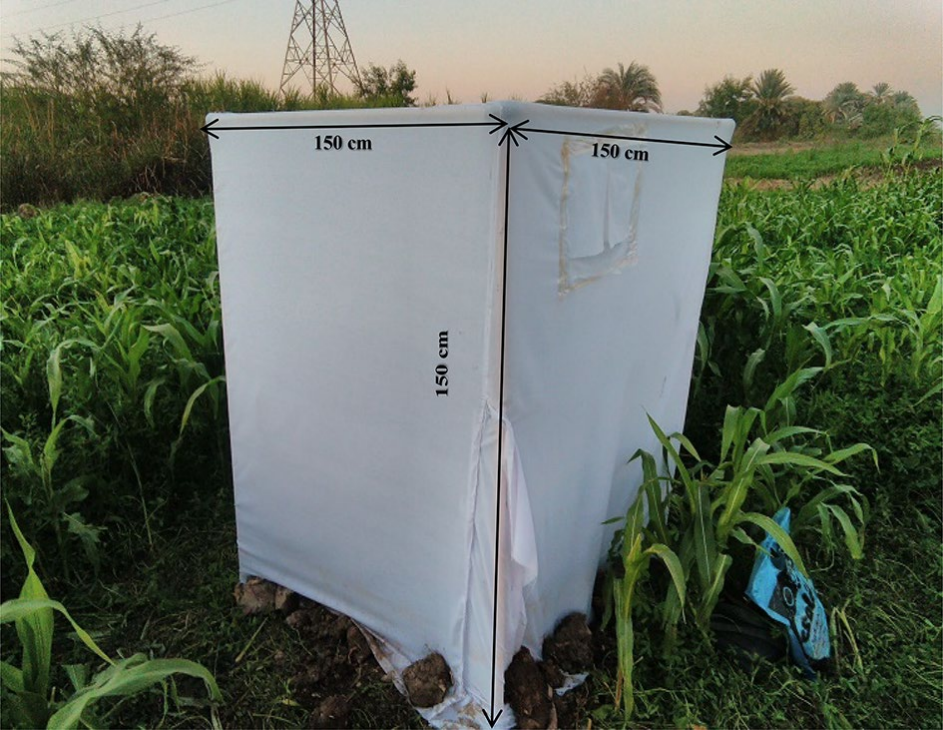
Semi-field cage: The cage includes a top cover, an entrance hatch, a transparent observation pane, a fabric-covered ventilation hole, a main tray, and a chiffon hemisphere for parasitoid performance. It also features an exit hatch, a hygrometer for temperature and humidity monitoring, and is covered with a white gauze cloth. The frame is constructed from plastic pipes, and the cage encloses maize plants for the experimental setup.

The semi-field experiment was conducted in a maize field free of infestation in the Kom-Ombo district, Aswan Governorate, Egypt (GPS: Latitude 24° 28’ 35” N, Longitude 32° 55’ 37” E) in September 2023 during the maize season. The mean temperature and relative humidity (RH) during the exposure of *Trichogramma* wasps to FAW eggs were 33.4° ± 1.12 °C and 66.5 ± 3.2%, respectively. A cage, constructed from plastic pipes and measuring 1.5 m³, was covered with a white gauze cloth to prevent insects from entering or exiting. This cage, placed in a maize field with 45-day-old plants, was used to test the efficiency of *T. evanescens* parasitism on FAW eggs. A hygrometer was attached inside each cage to monitor and record the mean temperature and RH. Each cage enclosed five maize plants, with three cages set up per day, repeated for three consecutive days. It is important to note that the cage was large enough to allow one person to enter for examination purposes (Fig. 2).

**Fig. 3:**
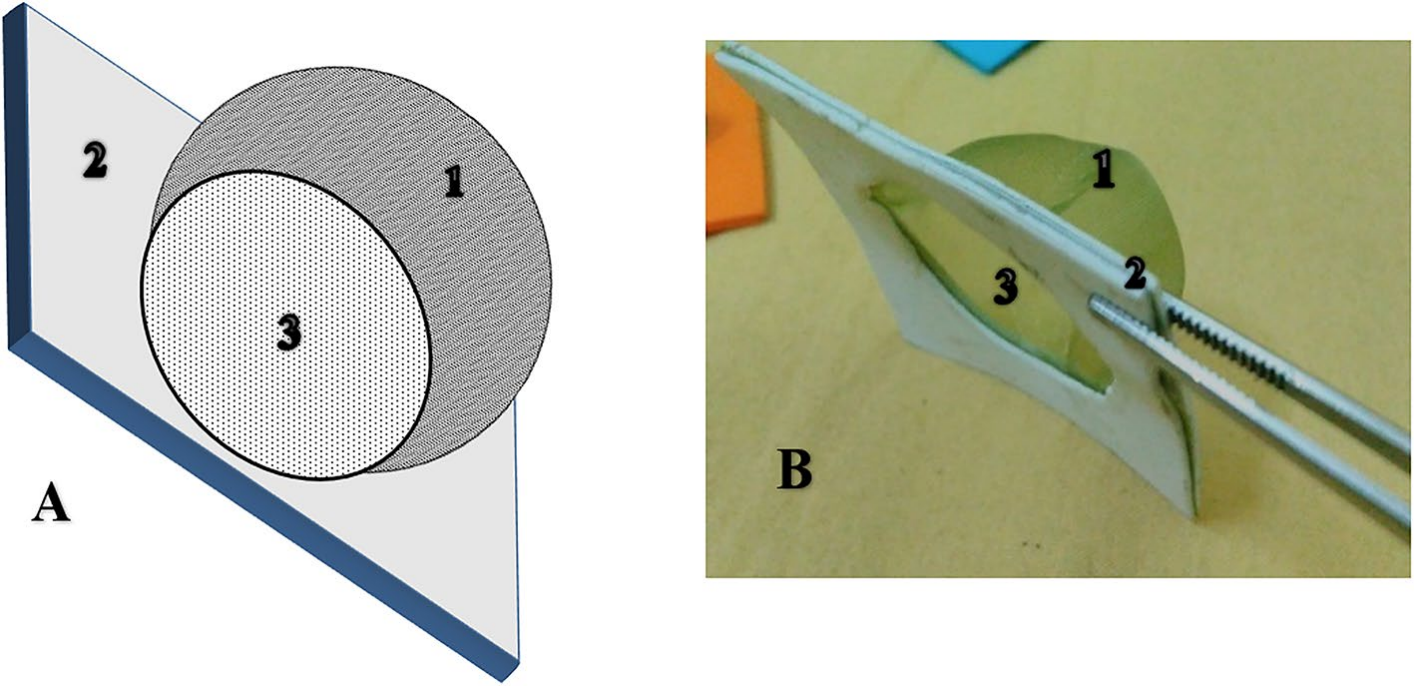
Hemisphere clip-cage (HCC), ((**A**) Diagram and (**B**) Photo): contain of: (1) a hemisphere (radius = 2 cm) of Micro-perforated polypropylene sleeve, (2) flat square of foam (2.5 cm^2^), and (3) Hole.

Egg masses were collected at 12-hour intervals from the FAW colony. These egg masses were suspended on corn leaves using a Hemisphere Clip-Cage (HCC) containing 200 parasitized *S. cerealella* eggs, which were expected to emerge within a few hours (Fig. 3). After one day^31^, the FAW egg masses were collected and transferred to plastic vials (2 *x*7 cm) under laboratory conditions with a temperature of 33.4 ± 1.12 °C and RH of 66.5 ± 3.2%. Daily observations were conducted to record and remove hatched larvae and to estimate parasitism rates.

### Hemisphere clip-cage (HCC)

A simple, lightweight clip-cage device was used for semi-field experiments with FAW eggs. The cage consists of two parts: a hemisphere (radius = 2 cm) made of micro-perforated polypropylene sleeve, glued with nontoxic adhesive to a flat square (2.5 cm^2^) of foam with a circular hole that fits the hemisphere’s radius, and another piece used for support. The micro-perforated polypropylene sleeve prevents predation by ants and other predators on *T. evanescens* and FAW eggs while allowing ventilation for the parasitoid adults (Fig. 3). The use of the Clip-cages went through many developments that went through various forms, beginning with heavy types^32–36^ and culminating in more lightweight and practical designs^37^.

### Statistical analysis

For statistical analysis, the number of FAW egg masses with scales was standardized to the lowest observed numbers, which were 11 for laboratory experiments and 31 for semi-field experiments. Similarly, the number of FAW egg masses based on layer count was standardized to the lowest observed numbers, which were 12 for laboratory experiments and 24 for semi-field experiments. A one-way ANOVA was performed using R (version 4.3.1/https://cran.r-project.org/bin/windows/base ), to determine if there were statistically significant differences among the various groups. The analysis considered factors such as the laboratory and semi-field observations under different setup conditions. The significance of results was assessed through the *p*-value, where a p-value less than 0.05 led to rejecting the null hypothesis of equal group means, indicating a significant difference in egg mass counts across groups.

## Results

### Efficiency of *T. evanescens* in laboratory

The data in Table 1 indicates that the mean number of eggs per mass was 179.9 ± 69.95 for egg masses without scales, 174.60 ± 57.27 for egg masses with average scales, and 244.57 ± 5.23 for egg masses with dense scales. Meanwhile, for egg masses with one, two, and three layers, the mean numbers were 122.57 ± 56.61, 201.07 ± 21.77, and 275.43 ± 13.99, respectively. The minimum number of eggs per mass was 41 in one-layer, scale-free egg masses, whereas the maximum number was 328 in three-layer, average-scale egg masses. Hatchability rates increased progressively, from 74.35% for non-scaled, to 81.25% for average-scaled, and 85.75% for dense-scaled egg masses. Similarly, hatchability rates ascended from 77.86% for single-layer, to 80.96% for double-layer, and 82.53% for triple-layer egg masses.

In addition, the results in Table 1 indicate that *T. evanescens* completed its life cycle on the FAW egg masses across all configurations, including variations in the presence of scales and the number of egg layers. However, the average parasitism rates and the mean number of *T. evanescens* parasitoids emerging from each egg mass were low. The presence of scales and the number of egg layers negatively impacted these outcomes.

As shown in Table 1, the absence of scales yielded the highest parasitism rate and number of parasitoids per egg mass, with a mean parasitism rate of 5.24% and an average of 6.90 parasitoids per egg mass. In contrast, egg masses with average scales had a parasitism rate of 3.09% and an average of 3 parasitoids per egg mass, while those with dense scales had a parasitism rate of 1.18% and an average of 3.07 parasitoids per egg mass.

**Table 1:** Mean no. of FAW eggs/mass, hatchability, mean no. of *T. Evanescens* parasitoids/mass and parasitism rates (± standard error) in laboratory.

Scales	Layer	No. of egg masses	No. of eggs/mass		Hatchability %		No. of parasitoids/mass		Parasitism %	
			Mean ± SE	Range	Mean ± SE	Range	Mean ± SE	Range	Mean ±SE	Range
Without	1	5	59.20 ± 6.29	41–80	68.85 ± 4.22	62.07–83.64	6.20 ± 2.33	0–14	10.15 ± 3.02	0-17.5
	2	4	179.00 ± 14.98	142–208	75.30 ± 2.12	69.01–77.98	5.00 ± 3.54	0–15	2.43 ± 1.70	0-7.21
	3	2	301.50 ± 43.50	258–345	78.91 ± 0.94	77.97–79.85	9.50 ± 1.50	8–11	3.14 ± 0.04	3.1–3.19
Mean of without scales			179.90 ± 69.95		74.35 ± 2.94		6.90 ± 1.35		5.24 ± 2.46	
Average	1	5	73.00 ± 8.47	45–96	78.18 ± 2.98	71.11–86.59	4.40 ± 1.91	0–9	6.58 ± 2.80	0-1-3.43
	2	5	179.60 ± 39.04	59–261	81.33 ± 2.63	75-89.27	3.60 ± 2.29	0–11	2.39 ± 1.78	0-9.17
	3	5	271.20 ± 27.81	200–328	84.24 ± 1.15	80.5-87.14	1.00 ± 0.70	0–5	0.31 ± 0.31	0-1.57
Means of average scales			174.60 ± 57.27		81.25 ± 1.75		3.00 ± 1.03		3.09 ± 1.84	
Dense	1	2	235.50 ± 27.50	208–263	86.55 ± 2.42	84.13–88.97	3.00 ± 1.90	0–6	1.14 ± 1.14	0-2.28
	2	5	244.60 ± 21.72	193–300	86.25 ± 0.60	84-88.97	3.20 ± 2.18	0–11	1.18 ± 0.78	0-3.85
	3	5	253.60 ± 12.60	218–293	84.44 ± 1.07	81.43–87.06	3.00 ± 1.90	0–9	1.23 ± 0.76	0-3.41
Means of dense scales			244.57 ± 5.23		85.75 ± 0.66		3.07 ± 0.07		1.18 ± 0.03	
Mean scales			199.69		80.45		4.32		3.17	
Mean of one layer			122.57 ± 56.61		77.86 ± 5.11		4.53 ± 0.93		5.96 ± 2.62	
Mean of two layers			201.07 ± 21.77		80.96 ± 3.17		3.93 ± 0.55		2.00 ± 0.41	
Means of three layers			275.43 ± 13.99		82.53 ± 1.81		4.50 ± 2.57		1.56 ± 0.83	
Mean layer			199.69		80.45		4.32		3.17	

Furthermore, egg masses with a single layer demonstrated better outcomes in terms of parasitism rates and the number of parasitoids per egg mass. The mean number of parasitoids per egg mass was 4.53, and the mean parasitism rate was 5.96%. In contrast, double-layer egg masses had mean values of 3.93 parasitoids per egg mass and a parasitism rate of 2%, while triple-layer egg masses exhibited 4.5 parasitoids per egg mass and a 1.56% parasitism rate.

**Fig. 4:**
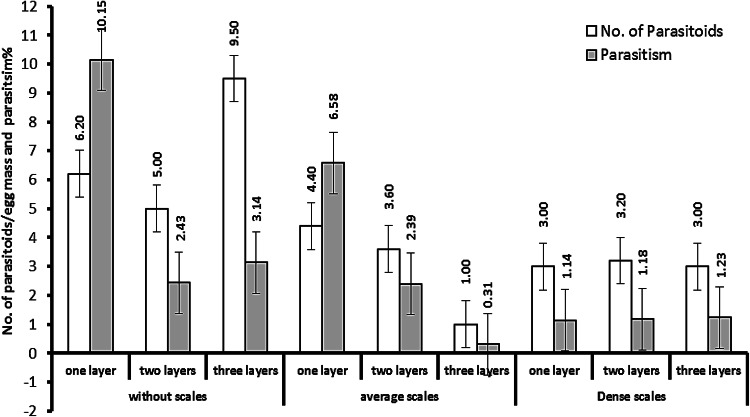
Efficiency of *T. evanescens* against FAW egg layers with different levels of scale dense in laboratory experiment.

Graphically illustrated in Fig. 4, the highest parasitism rate, 10.15%, was achieved in single-layer egg masses without scales. Conversely, Conversely, the highest mean number of parasitoids per egg mass, 9.50, was observed in triple-layer egg masses without scales, while the lowest number, 5.00, was recorded in two-layer masses. For egg masses with average scales, the highest mean was 4.40 for single layers, and the lowest was 1.00 for three layers. In the case of dense scales, there were no significant differences in parasitoid numbers across the layers. These values reflect the emergence of *T. evanescens* offspring from the egg masses, highlighting the influence of structure and scale density on the successful emergence rates of progeny.

### Efficiency of ***T. evanescens*** in semi-field experiment

**Fig. 5:**
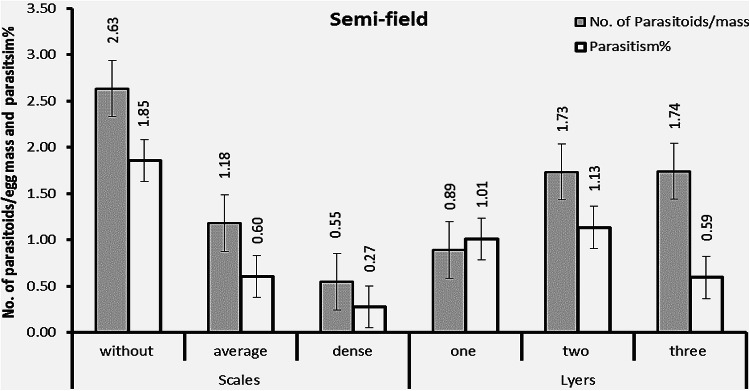
Efficiency of *T. evanescens* against FAW egg masses with varying layers and scale densities in a semi-field experiment.

Data presented in Table 2 and illustrated in Fig. 5 show that *S. frugiperda* egg masses parasitized by *Trichogramma* under field conditions are rare. Both the mean number of *T. evanescens* parasitoids emerging from each egg mass and the average parasitism rates were low, influenced by the presence of scales and the number of egg layers. Parasitism rates and the number of parasitoids per mass in egg masses without scales were higher compared to those with average or dense scales. The recorded averages were 2.63 parasitoids per mass and a parasitism rate of 1.85% for egg masses without scales; 1.18 parasitoids per mass and a parasitism rate of 0.60% for those with average scales; and 0.55 parasitoids per mass and a parasitism rate of 0.27% for those with dense scales.

**Table 2:** Semi-field evaluation of *T. evanescense* parasitism on FAW, *S. frugiperd* egg masses (mean no. of FAW eggs/mass, hatchability, mean no. of *T. Evanescens* parasitoids/mass and parasitism rates (± standard error) in semi-field experiment.

Scales	Layer	No. of egg masses	No. of eggs/mass		Hatchability %		No. of parasitoids/mass		Parasitism %	
			Mean ± SE	Range	Mean ± SE	Range	Mean ± SE	Range	Mean ± SE	Range
Without	1	17	87.88 ± 2.23	69–100	86.68 ± 0.80	80.52–92.74	2.47 ± 0.42	0–5	2.80 ± 0.48	0-5.43
	2	7	154.14 ± 10.26	115–186	90.37 ± 1.08	86.9-94.09	2.43 ± 0.78	0–5	1.78 ± 0.63	0-4.35
	3	7	297.14 ± 15.38	210–330	91.63 ± 0.39	90.43–93.24	3.00 ± 1.07	0–6	0.99 ± 0.35	0-2.03
Mean of without scales			179.72 ± 61.75		89.56 ± 1.49		2.63 ± 0.18		1.85 ± 0.52	
Average	1	5	94.40 ± 2.46	89–100	91.02 ± 0.91	88.76-94	0.20 ± 0.20	0–1	0.22 ± 0.22	0-1.12
	2	24	182.17 ± 2.81	125–290	90.89 ± 0.12	87.2-94.42	1.71 ± 0.12	0–5	1.01 ± 0.07	0-3.52
	3	11	289.27 ± 14.67	198–362	93.04 ± 0.35	91.02–95.07	1.64 ± 0.51	0–5	0.58 ± 0.19	0-1.86
Means of average scales			188.61 ± 56.35		91.65 ± 0.70		1.18 ± 0.49		0.60 ± 0.23	
Dense	1	3	96.00 ± 3.51	89–100	92.80 ± 1.39	90.91–95.51	0.00	0.00	0.00	0.00
	2	17	193.29 ± 12.70	129–315	92.44 ± 0.38	87.6-94.98	1.06 ± 0.38	0–4	0.61 ± 0.22	0-2.33
	3	12	284.67 ± 11.55	180–329	93.94 ± 0.41	91.67–96.21	0.58 ± 0.31	0–3	0.22 ± 0.12	0-1.11
Means of dense scales			191.32 ± 54.47		93.06 ± 0.45		0.55 ± 0.31		0.27 ± 0.18	
Mean scales			186.55		91.42		1.45		0.91	
Mean of one layer			92.76 ± 2.48		90.17 ± 1.82		089 ± 0.79		1.01 ± 0.90	
Mean of two layers			176.53 ± 11.65		91.23 ± 0.62		1.73 ± 0.40		1.13 ± 0.34	
Means of three layers			290.36 ± 3.64		92.87 ± 0.67		1.74 ± 0.70		0.59 ± 0.22	
Mean layer			186.55		91.42		1.45		0.91	

## Discussion

The study sheds light on the various factors influencing the parasitism efficiency of *Trichogramma evanescens* on FAW egg masses. Previous research by Hou et al.^6^. and Mohamed et al.^38^. has shown that the thickness of egg mass scales and the number of layers significantly impact parasitism rates. Hou et al. discovered that strategic timing of parasitoid releases—aimed at periods when scales naturally thin—could enhance control success, with the highest parasitism observed in egg masses at Level I scale thickness. Mohamed et al. emphasized environmentally friendly strategies, highlighting that single-layer egg masses exhibited the highest parasitism rates, which underlines the need for targeted biological control efforts.

Building on these findings, Li et al.^39,40^. further elucidated that egg protection strategies, such as scale formation, initially hinder parasitism but present opportunities for improved biocontrol once scales thin. They suggest incorporating protective trait insights into control programs to manage invasive pests more effectively. These studies collectively underscore the necessity of a multifaceted approach for biological control, which aligns with our own findings on the influence of egg mass structure on parasitism dynamics.

The parasitism efficiency of various *Trichogramma* species against *Spodoptera frugiperda* (FAW) egg masses has been extensively studied across different geographical regions and under diverse agricultural practices. Our results in Aswan Governorate, Upper Egypt, alongside studies from Guanajuato, Central Mexico, and other regions, provide compelling insights into the factors influencing the success of these biological control agents^41^.

In Guanajuato, a study involving over 4,000 sentinel egg masses of *S. frugiperda* revealed the exclusive recovery of *Trichogramma atopovirilia* from three locations. Historically, *T. atopovirilia*, known from the southern United States to Brazil, has been documented primarily parasitizing *Diatraea* species eggs across coastal states in Mexico^41^. Its parasitism in *S. frugiperda* eggs was rarely reported, making this study the first confirmed presence of *T. atopovirilia* in Central Mexico’s maize and sorghum fields^27,42–44^. This emphasizes the potential for applying tailored parasitoid management strategies across different pest and crop scenarios, as demonstrated by Iqbal et al.^1^ with their findings on optimizing Trichogramma ostriniae emergence through specific age and species ratios.

Despite extensive releases of *Trichogramma pretiosum* for controlling *S. frugiperda* in maize and vegetables, this parasitoid was not recovered from the studied fields in Guanajuato. Several reasons could explain this. The releases might not have led to successful establishment^42^. Although the study sites avoided insecticide usage, surrounding areas may have affected parasitoid recovery adversely, given the known lethal and sublethal effects of insecticides on crops^45^. Moreover, *S. frugiperda* populations peak early in the crop cycle, suggesting future collections should target March, April, August, and September when egg laying is most abundant. Conversely, *T. atopovirilia* recoveries peaked in September and October of consecutive years, highlighting its resilience and effectiveness under the given conditions.

Generalist predators were predominant in these fields, and crop management practices, such as the presence of weeds, substantially influenced predation rates^46^. For example, fields in Cortazar with traditional management showed higher predation rates (> 21%) compared to Roque, which employs conventional, weed-free management. These findings underscore the importance of conserving natural habitats and predator-friendly practices within agricultural systems. Previous studies have also observed similar patterns, indicating that structures like weeds serve as shelters and food sources, thereby increasing predator abundance in non-weed-controlled plots^27,47,48^.

In the present study, the parasitism efficiency of *Trichogramma evanescens* on FAW egg masses, focusing on physical characteristics like scales and egg layers. This study found that egg masses with fewer scales and layers exhibited higher parasitism rates. For instance, single-layer, scale-free egg masses had the highest parasitism (10.15%) and parasitoid emergence rates. Dense scales significantly deterred parasitism, evidenced by a marked reduction in parasitism rates and emerging parasitoids in such masses. Single-layer egg masses were more susceptible to parasitism than multiple-layer masses, reinforcing the role of egg mass structure in parasitism dynamics. These observations align with the hypothesis that physical barriers present a challenge for parasitoids.

Field studies showed that despite high-dose releases, parasitism rates remained low due to environmental variables like adverse weather and lack of natural infochemicals essential for host location. Semi-field conditions also yielded lower parasitism rates, affected by fluctuating temperatures, humidity, and the presence of competing species. Similar challenges have been documented in previous researches, highlighting the intricate arrangement of FAW eggs in multiple layers and the presence of scales as significant obstacles to parasitoid action^49–52^.

The comprehensive study of *T. evanescens* and findings from Guanajuato highlight the significant role of egg mass physical traits and environmental factors in the efficacy of parasitoids^41^. These insights suggest a two-pronged approach for enhancing biological control strategies. First, timing releases to when FAW eggs are most vulnerable and ensuring conditions that favor parasitoid establishment, like maintaining natural habitats. Second, developing and releasing *Trichogramma* strains capable of overcoming physical and environmental barriers posed by FAW egg masses.

Previous studies by researchers like Beserra et al.^53^. and Goulart^54^ have documented similar challenges, highlighting the intricate arrangement of FAW eggs in multiple layers and the presence of scales as significant obstacles to parasitoid action. Thus, the current findings are in line with established entomological research, adding robustness to the study’s conclusions^55,56^.

In this regard, the egg parasitoid *Trichogramma* spp. (Hymenoptera: Trichogrammatidae) is employed across multiple countries to manage lepidopteran pests through wide-scale release over millions of hectares of valuable agricultural land^57–59^.

To satisfy the demand for biological pest control in crops such as maize, soybean, cotton, tomato, and sugarcane, several companies specialize in the mass production of these parasitoids. In Brazil alone, four biofactories are officially registered in the Phytosanitary Agrochemicals System for producing *Trichogramma* spp^60,61^. Within this genus, *T. pretiosum* Riley (Hymenoptera: Trichogrammatidae) is marketed specifically for controlling lepidopteran eggs and has been identified in around 18 different host species and 13 types of crops^62–65^.

In Turkey, deploying 120,000 *Trichogramma* parasitoids per hectare to manage *H. armigera* in cotton fields led to a 52.5% reduction in parasitism frequency from its original level^66^. This underscores the promise of using *Trichogramma* in the biological control of H. armigera.

Overall, this research emphasizes the necessity for a multifaceted and sustained approach in biological control programs, combining the strengths of different *Trichogramma* species, optimizing agricultural practices for conservation, and advancing scientific understanding to adapt to varying ecological contexts.

## Conclusions

An experiment was conducted to evaluate the capacity of the local parasitoid, *T. evanescens*, to parasitize Fall Armyworm (FAW) egg clusters, especially considering that Egypt hosts several laboratories dedicated to producing *T. evanescens* for managing *Chilo agamemnon*. The results clearly indicated that the parasitism rates of *T. evanescens* on FAW egg masses were suboptimal under both laboratory and semi-field conditions. Even without scales and layers, only 6.2 and 2.47 parasitoids emerged from populations of 59.2 and 87.88 FAW eggs, with corresponding parasitism rates of 10.15% and 2.8% in laboratory and semi-field settings, respectively. While these figures primarily reflect parasitoid emergence, further analysis involved dissecting host eggs to verify actual parasitism occurrences. This analysis confirmed that parasitism extended beyond what emergence data alone suggested. Additionally, a reduction in FAW egg hatching rates due to parasitoid attacks was observed, offering a comprehensive perspective on the impacts of parasitoid activity on pest populations. These findings imply that the presence of scale barriers and multiple egg layers significantly impedes the parasitism efficiency of T. evanescens. Nonetheless, recognizing these limitations is a crucial step towards refining biological control strategies against the Fall Armyworm in Egypt.

## Data Availability

All data generated or analyzed during this study are included in this manuscript.
